# Does cyclic water stress damage wheat yield more than a single stress?

**DOI:** 10.1371/journal.pone.0195535

**Published:** 2018-04-09

**Authors:** Jinfeng Ding, Zhengjin Huang, Min Zhu, Chunyan Li, Xinkai Zhu, Wenshan Guo

**Affiliations:** 1 Jiangsu Key Laboratory of Crop Genetics and Physiology, Yangzhou University, Yangzhou, China; 2 Co-Innovation Center for Modern Production Technology of Grain Crops, Yangzhou University, Yangzhou, China; 3 Wheat Research Institute, Yangzhou University, Yangzhou, China; 4 Institute of Botany, Jiangsu Province and Chinese Academy of Sciences (Nanjing Botanical Garden Mem. Sun Yat-Sen), Nanjing, China; 5 Institutes of Agricultural Science and Technology Development, Yangzhou University, Yangzhou, China; 6 Joint International Research Laboratory of Agriculture and Agri-Product Safety of Ministry of Education of China, Yangzhou University, Yangzhou, China; University of Tasmania, AUSTRALIA

## Abstract

The occurrence of water stress during wheat growth is more frequent due to climate change. Three experiments (cyclic drought, cyclic waterlogging, and cyclic drought plus waterlogging) were conducted to investigate the effects of mild and severe cyclic/single water stress at elongation and heading stages on winter wheat (*Triticum aestivum* L.) yield. The effect of either mild drought at elongation or mild waterlogging at heading on wheat yield was not significant; however, significance did occur under other single water stresses. As the stress becomes more severe, the yield loss significantly increases. Extreme drought/waterlogging treatment at elongation caused a greater yield penalty than stress at heading stage. Except the combination of mild drought and mild waterlogging treatment, cyclic water stress significantly decreased wheat yields. The decrease in wheat yield under cyclic severe drought and waterlogging was significantly higher than any other treatment, with percentage decreases of 71.52 and 73.51%, respectively. In general, a yield reduction from mild cyclic water stress did not indicate more severe damage than single treatments; in contrast, grain yield suffered more when water stress occurred again after severe drought and waterlogging. Drought during elongation significantly decreased kernel number, whereas drought at heading/waterlogging during elongation and heading decreased the spike weight, which might be the main reason for the yield penalty. Furthermore, water stress caused variation in the decrease of total biomass and/or harvest index. The present study indicates comprehensive understanding of the types, degree, and stages of water stress are essential for assessing the impact of multiple water stresses on wheat yield.

## Introduction

Water stress has become one of the major constraints on wheat production. Extreme weather is occurring more frequently due to climate change, including changes in precipitation patterns. Consequently, the dry areas become drier and wet areas are wetter, resulting in wheat exposed to more than one water stress condition during the wheat-growing season [1, 2]. Furthermore, very different degrees of water availability occur, leading to alternate occurrences of drought and waterlogging stress. The middle and lower reaches of the Yangtze River are the main production areas of rice and wheat in China, where more than 12% of the wheat-planting area of China is located [3]. According to meteorological and production statistics from Jiangsu province, in this area, drought stress occurs every three years, and waterlogging stress occurs every two years, causing more than 10% yield losses; these two stress types mostly occur in spring [3].

It has been reported that wheat is more sensitive to drought stress from stem elongation to the milking stage [4], whereas Eck [5] argued that the critical period occurred during the tillering and jointing stages. In general, the reproductive and grain-filling phases are regarded to be the most sensitive stages to drought stress [6]. Opinions on which stage is most sensitive to waterlogging stress were similar to the conclusions on the aforementioned drought stress. Araki et al [7] indicated that the highest yield reduction was found under post-anthesis waterlogging rather than during the elongation stage, whereas de San Celedonio et al. [8] observed that the highest yield penalties occurred when waterlogging occurred between stem elongation and anthesis. Setter and Waters [9] suggested that wheat is least tolerant to waterlogging at the pre-emergence, seedling growth and reproductive stages.

At the same time, the effects of water stress on wheat depends on the duration and severity of the stress. Ercoli et al. [10] observed that grain yield, dry matter accumulation and remobilization were negatively affected by drought stress during the grain-filling stage. Ma et al. [11] reported that extreme stress caused more yield decrease than moderate stress. Mild drought stress at the grain-filling stage, however, could promote remobilization of carbon assimilates to the grains, accelerating grain filling and, ultimately, improve the yield [12]. Mild water stress (65–70% water field capacity) was found under conditions of water limitation [13]. These findings indicated that the effect of the waterlogging intensities on wheat are relatively lower, possibly because waterlogging is not dependent on where surface drains occur [14]. Except for the aforementioned stages and severity, the effects of water stress on wheat also depend on soil type and environmental conditions [9, 15, 16], cultivars grown [17, 18], and cultivation technologies [12, 19].

Many studies have been devoted to evaluating the effects and mechanisms of water stress on wheat [20–22], whereas the adopted experimental methods in previous studies mainly focused on a single stress during the growth period, and few studies paid attention to the responses and mechanisms of wheat to multiple water stresses or on the differences between single and multiple treatments. Cycles or intermittent water stress is thought to approximate natural conditions better than single treatments [23]. Several publications have demonstrated the effects of cyclic drought stress on wheat cultivars [23, 24] and the effects of intermittent waterlogging on wheat [25]. Dickin and Wring [26] investigated the effects of winter waterlogging and summer drought on the growth and yield of winter wheat. The objective of the current study is to investigate the effects of mild and severe degrees of cyclic or a single water stress event at the elongation and heading stages of winter wheat (*Triticum aestivum* L.) yield. Furthermore, the differences in yield components, total biomass and harvest index (HI) between the different treatments were analyzed. The results will provide important information for developing approaches to irrigation and drainage regulation for stress-relief and high and stable production of wheat.

## Materials and methods

### Plant materials and growth conditions

The experiments were conducted at the Agricultural Experiment Station (32°39′E, 119°42′N) of the Agricultural College of Yangzhou University in China during the growth seasons of 2013–2014 (2013) and 2014–2015 (2014). A winter wheat (*Triticum aestivum* L.) cultivar (Yangmai 20) widely grown in the Yangtze River Basin of China was selected.

A pot test was performed under natural conditions of light and temperature. Each PVC pot was 26-cm wide at the top, 18-cm wide at the bottom, and 26-cm deep. Before filling, fine soil was prepared for each container by sieving through 5-mm mesh and then mixed with the following fertilizers: 3.6 g pot^-1^ inorganic compound fertilizer (containing 15% N, 15% P_2_O_5_, and 15% K_2_O) and 0.83 g pot^-1^ urea (containing 46% N). In addition, 0.42 g pot^-1^ urea was dressed at the four-leaf stage, and 3.6 g pot^-1^ inorganic compound fertilizer and 0.83 g pot^-1^ urea was dressed at elongation. Each container was filled with 11 kg of soil and was then watered at the same rate of 5 L to compact the soil. After the seeds were sown in each container, an additional 1 kg of soil was used to cover them. Twelve seeds were sown in each pot on November 3 during the two years. At the three-leaf stage, the plants were thinned to six plants per pot. The experiments were conducted without biotic stresses, and all the weeds were removed by hand.

The soil was loamy clay and contained 14.03 g kg^-1^ organic matter, 78.06 mg kg^-1^ alkali hydrolysable N, 35.48 mg kg^-1^ Olsen-P, and 81.86 mg kg^-1^ exchangeable K in 2013, and 9.62 g kg^-1^ organic matter, 79.95 mg kg^-1^ alkali hydrolysable N, 38.52 mg kg^-1^ Olsen-P, and 85.37 mg kg^-1^ exchangeable K in 2014 before fertilizer application.

### Experimental design

The experiments were divided into three parts, including a cyclic drought assay in 2013, a cyclic waterlogging assay in 2014, and a cyclic drought and waterlogging assay in 2014. All three experiments utilized a randomized complete block design of two factors and three levels, with 10 replicates. Plants were grown under a relative soil moisture content (SMC) of approximately 75% before and after treatments. A transparent waterproof canopy at 3 m height with proportional light transmission of 75±10% was used to avoid the rainfall influence.

### Cyclic drought experiment

The SMC of all pots was controlled 10 days before application of treatments to gradually achieve three expected values: approximately 75% SMC (control), approximately 60% SMC (mild drought), and approximately 40% SMC (severe drought). The first cycle of treatments was applied for 12 days at elongation (from March 29^th^ to April 9^th^). After the first treatment, the plants were re-watered to control the SWC to approximately 75% and were then subjected to the second run treatments. Pots with the same SMC in the first cycle treatments were divided into three groups: approximately 75% SMC (the control), 60% SMC (mild drought), and 40% SMC (severe drought). The second-cycle treatments were applied for 12 days at heading (from April 16^th^ to April 27^th^).

### Cyclic waterlogging experiment

All the pots were divided into three SMC groups: approximately 75% SMC (Control), 90% SMC (mild waterlogging), and 100% SMC (approximately 2 cm water aboveground, representing severe waterlogging). The first cycle of treatments was applied for 12 days at elongation (from March 17 to March 28). After the first treatment, the pots were drained to control (the SMC is around 75%), and were then subjected to the second run treatment. Each group of pots was again divided into three groups, that is approximately 75% SMC (control), 90% SMC (mild waterlogging), and 100% SMC. The second cycle treatments lasted for 12 days at heading (from April 10 to April 21).

### Cyclic drought and waterlogging experiment

All the pots were divided into three SMC groups: approximately 75% SMC (control), 60% SMC (mild drought), and 90% SMC (mild waterlogging). The first cycle of treatments lasted for 12 days at elongation (from March 17 to March 28). After the treatment, the pots were re-watered or drained to control level (approximately 75%) and then subjected to the second run. Again each group was divided into three groups: approximately 75% SMC (Control), 60% SMC (mild drought), and 90% SMC (mild waterlogging). The second treatment was applied for 12 days at heading (from April 10 to April 21).

**Fig 1:**
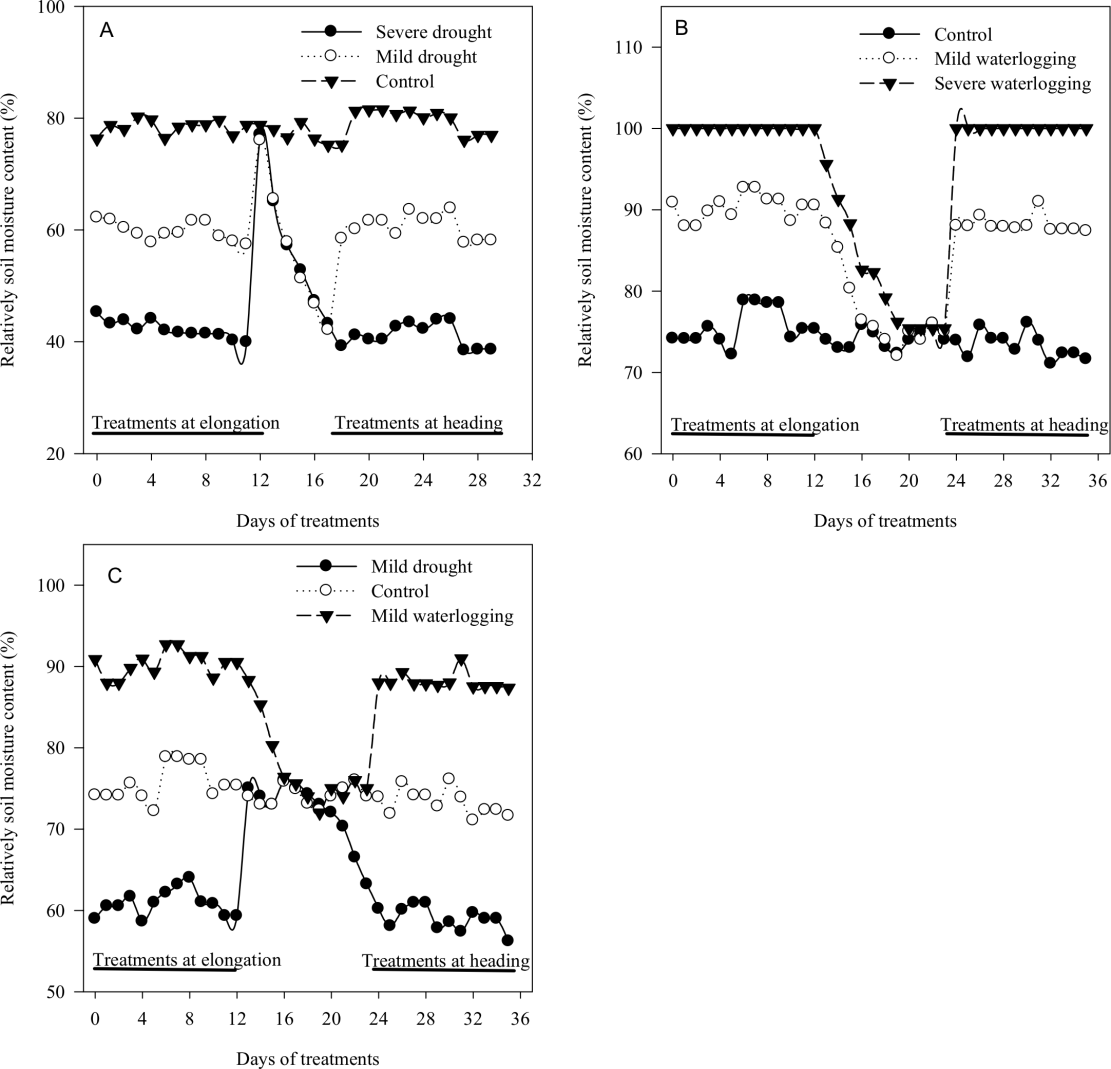
Relative soil moisture content during cyclic drought (A), cyclic waterlogging (B), and cyclic drought with waterlogging (C) experiments.

The SMC in the pots was calculated according to the soil water content and field water capacity. The soil water content in the pots was measured using the weighing method. The SMCs are shown in Fig 1. Irrigation amount was calculated as follows:

$$Irrigation\;amount\;\left(g\;pot^{‑1}\right)=\left(the\;desired\;SMC\times field\;water\;capacity\right)\times soil\;dry\;weight‑water\;weight‑plant\;weight$$

Where plant weight was measured every 10 days to eliminate the influence of plant growth. The pots were watered twice a day, at 09:00 and 15:00, with a given amount of water to bring the water concentration of the pots to a desired level by weighing the pots individually.

### Sampling and measurements

The plants were harvested at maturity. The spikes per plant and kernels per spike were counted. And the total biomass of aboveground plants was determined after drying at 70°C to a constant weight. A total of 1000 kernels were randomly counted and weighed. The grain yield value was adjusted to 13% moisture. The harvest index (HI) was calculated as follows:

Harvestindex=Dryweightofgrains/Totalabovegrounddryweightatmaturity

### Statistical analysis

The design for each of the three experiments was a factorial experiment with two factors and arranged in a completely randomized design with 10 replications for each treatment. The data of each variable were subjected to analysis of variance (ANOVA) with the DPS 7.05 statistical package, according to this design. When F values were significant, means were separated by the LSD test (*P* ≤ 0.05).

## Results

### Effects of cyclic and single drought stress on yield, yield components, total biomass, and HI

As shown in Table 1, drought stress significantly decreased the grain yield, either at elongation or at heading, as well as the kernels per spike, 1000-kernel weight, total biomass, and HI. Furthermore, the spikes per plant were significantly affected by drought stress at elongation. Interaction effects occurred between the two stages of treatment on grain yield and HI.

**Table 1:** The ANOVA table for yield, yield components, total biomass, and HI under drought stress at stages of elongation and heading.

Source of variation	Grain yield	Spikes per plant	Kernels per spike	1000-kernel weight	Total biomass	HI
Drought stress at elongation (DSE)	164.7**	471.9**	44.3**	142.9**	91.5**	95.2**
Drought stress at heading (DHE)	153.5**	3.6	22.6**	31.8**	66.8**	59.3**
DSE×DHE	12.1**	0.9	3.0	2.2	0.9	8.2**

Values in the table are F-values.

** Significant difference at P≤0.01.

#### Yield and yield components

**Fig 2:**
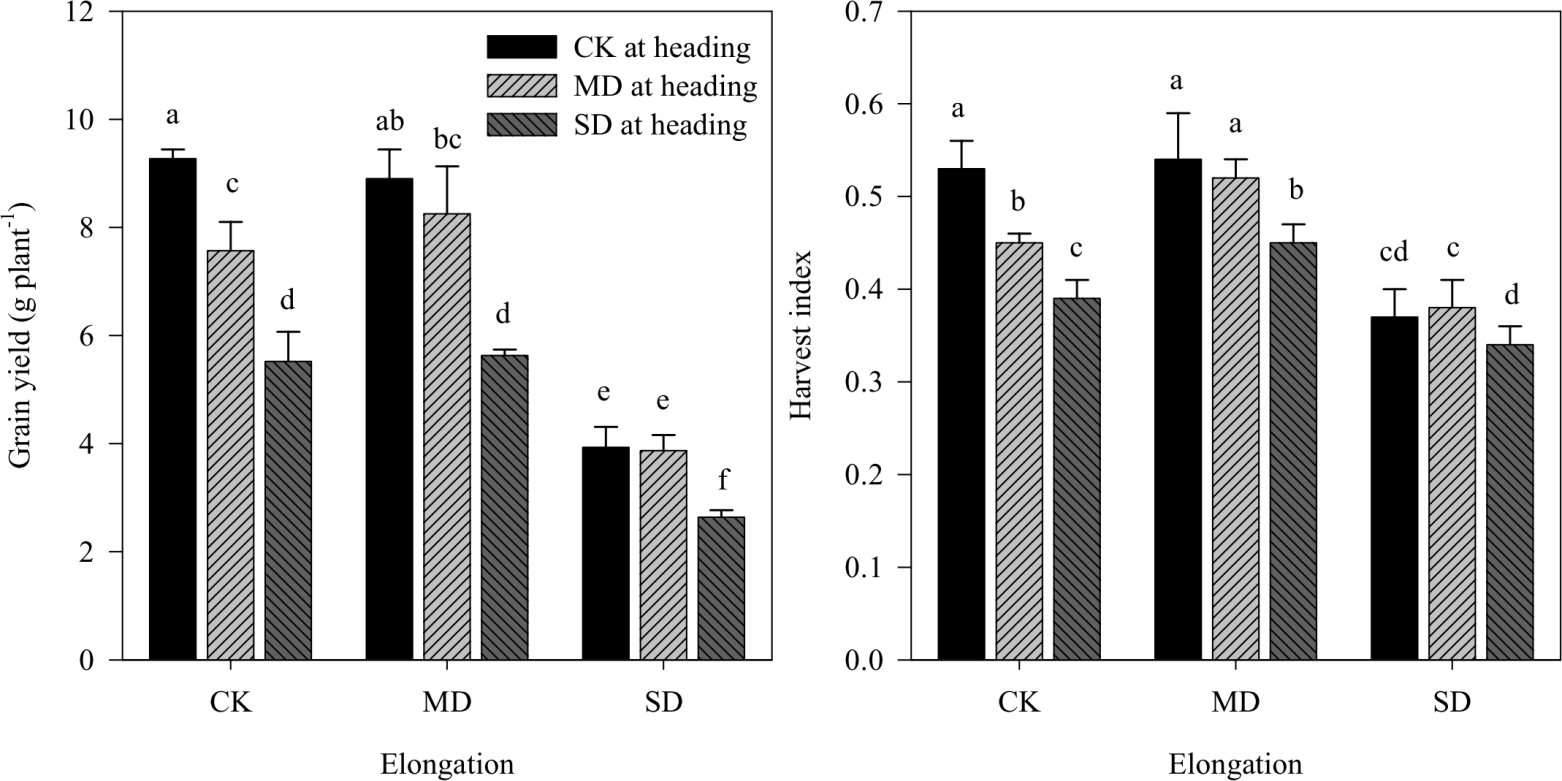
Effects of drought stress during the stages of elongation and heading on grain yield and HI. CK, MD and SD denote control, mild drought, and severe drought, respectively. Different letters indicate statistical significance among treatments at the *P*≤0.05 level.

Mild drought at elongation had no significant influence on grain yields because the increase in 1000-kernel weight compensated for the loss of spikes per plant and kernels per spike (Table 2). Nonetheless, severe drought significantly decreased the grain yield, mainly due to the decrease in spikes per plant and kernels per spike, notwithstanding the 1000-kernel weight increase. Various degrees of drought at heading, however, significantly decreased the grain yield due to the loss of spike weight. When the drought stress became severe, the loss of grain yield, kernels per spike and 1000-kernel weight increased. The results indicated that grain yield losses significantly decreased under drought treatment at the heading stage after the first drought treatments at elongation (Fig 2).

**Table 2:** Yield, yield components, total biomass, and HI as affected by various degrees of drought stress.

Treatments	Grain yield (g plant^-1^)	Spikes per plant	Kernels per spike	1000-kernel weight (g)	Total biomass (g plant^-1^)	HI
Drought stress at elongation						
Control	7.45a	4.20a	46.90a	37.56c	14.46a	0.46b
Mild drought	7.59a	3.90b	44.73a	43.11b	13.48a	0.50a
Severe drought	3.48b	2.07c	36.02b	46.32a	8.47b	0.36c
Drought stress at heading						
Control	7.37a	3.42a	45.81a	46.09a	13.39a	0.48a
Mild drought	6.56b	3.51a	44.49a	42.31b	12.79a	0.45b
Severe drought	4.59c	3.24a	37.35b	38.59c	10.22b	0.39c

Different letters indicate statistical significance at the *P*≤0.05 level among different treatments in the same stages.

Compared with the control values, the decreased yield under the single mild drought at elongation was not significant; on the contrary, the single mild drought at heading significantly decreased the grain yield, by 18.34%. The single severe drought at elongation and at heading significantly decreased the grain yield, by 57.61 and 40.45%, respectively. The decrease in grain yield only under the combinations including severe drought at elongation was significantly higher than the single treatments. Across all the treatments, the grain yield under a combination of severe drought at elongation and heading was lowest, being only 28.48% of the control treatments.

#### Total biomass and HI

As shown in Table 2 and Fig 2, total biomass was not significantly affected by mild drought, but was greatly reduced under the severe drought treatment at either the elongation or the heading stage. Interestingly, mild drought stress significantly increased HI while the extreme drought treatment during elongation significantly decreased HI. HI significantly decreased under drought at the heading stage, and its decrease increased with degree of stress. The HI decrease was diminished under drought at heading, after the first drought treatments at elongation.

### Effects of cyclic and single waterlogging stress on yield, yield components, total biomass, and HI

As shown in Table 3, waterlogging stress significantly affected grain yield, either at elongation or at heading, as well as kernels per spike, 1000-kernel weight, and total biomass. Furthermore, spike number per plant and HI were significantly affected by waterlogging stress at elongation. An interaction effect occurred between the two stages of treatment for grain yield, kernels per spike, 1000-kernel weight, and total biomass.

**Table 3:** The ANOVA table for yield, yield components, total biomass, and HI under waterlogging stress at stages of elongation and heading.

Source of variation	Grain yield	Spikes per plant	Kernels per spike	1000-kernel weight	Total biomass	HI
Waterlogging stress at elongation (WSE)	380.1**	77.8**	25.3**	277.0**	46.7**	10.5*
Waterlogging stress at heading (WHE)	94.4**	2.4	48.0**	34.8**	34.0**	0.7
WSE×WHE	20.2**	0.9	18.4**	10.8**	12.5**	0.1

Values in the table are *F*-values.

* Significant difference at *P*≤0.05.

** Significant difference at *P*≤0.01.

#### Yield and yield components

Grain yield significantly decreased under waterlogging, either at elongation or at heading, and its decrease increased with degree of stress (Table 4). The reasons for the grain yield loss, however, were different among various waterlogging treatments. Spike number per plant was not impacted by mild waterlogging stress, while this treatment significantly decreased the spike weight; the same trend could be observed under the treatment of severe waterlogging stress during heading. Severe waterlogging stress during elongation not only significantly decreased spike weight but also restricted spike number per plant. Further analysis indicated that a minimal decrease in grain yield, kernels per spike, and 1000-kernel weight occurred under mild waterlogging at heading in the primed plant of the control, whereas a drastic decrease occurred under severe waterlogging at heading in the primed plant, but remained minimal under mild waterlogging.

**Table 4:** Yield, yield components, total biomass, and HI as affected by various degrees of waterlogging stress.

Treatments	Grain yield (g plant^-1^)	Spikes per plant	Kernels per spike	1000-kernels weight (g)	Total biomass (g plant^-1^)	HI
Waterlogging stress at elongation						
Control	8.10a	3.82a	47.21a	45.11a	18.53a	0.39a
Mild waterlogging	6.79b	3.80a	42.05b	42.28b	17.65a	0.34b
Severe waterlogging	4.47c	3.36b	36.87c	34.51c	12.52b	0.32b
Waterlogging stress at heading						
Control	7.26a	3.73a	44.67a	43.24a	17.89a	0.36a
Mild waterlogging	6.78b	3.70a	43.71a	41.60a	17.11a	0.35a
Severe waterlogging	5.32c	3.55a	37.74b	37.05b	13.70b	0.34a

Different letters indicates statistical significance at the *P*≤0.05 level among different treatments at the same stages.

**Fig 3:**
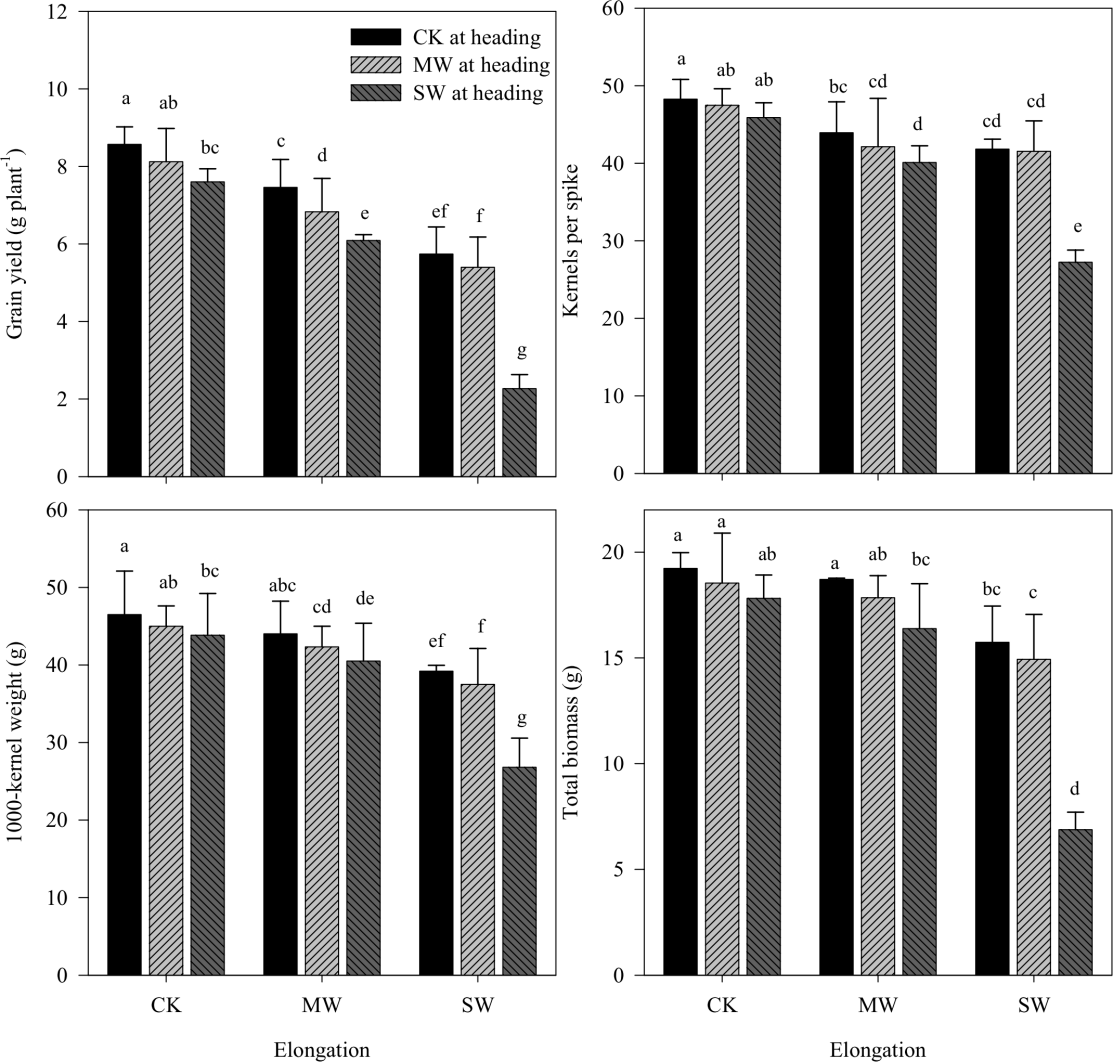
Effects of waterlogging stress at the elongation and heading stages on grain yield, kernels per spike, 1000-kernel weight and total biomass. CK, MW and SW denote control, mild waterlogging, and severe waterlogging, respectively. Different letters indicate statistical significance at the *P*≤0.05 level among different treatments.

Compared to the control values, the mild waterlogging treatment during elongation significantly decreased grain yield, by 12.95%; in contrast, single mild waterlogging at heading did not decrease the grain yield (Fig 3). Furthermore, single severe waterlogging at elongation and at heading significantly decreased grain yield, by 33.02 and 11.32%, respectively. The decreases in grain yield under the cyclic waterlogging treatments were much less than under the single treatments except for under the combination of severe waterlogging and mild waterlogging. Across all treatments, the grain yield under the combination of severe waterlogging at elongation and heading was lowest, being only 26.49% of the control treatments.

#### Total biomass and HI

As shown in Table 4 and Fig 3, mild waterlogging stress did not significantly reduce the total biomass and HI, while the mild waterlogging stress during elongation significantly impacted the HI. Severe waterlogging stress significantly decreased the total biomass and HI, except for HI under severe waterlogging at heading. Further analysis showed that minimal biomass loss occurred under mild waterlogging at heading in the primed plant of the control, but its loss was drastic under severe waterlogging at heading in the primed plant with severe waterlogging and was still minor under mild waterlogging.

### Effects of cyclic and single drought/waterlogging stress on yield, yield components, total biomass, and HI

As shown in Table 5, water stress at either the elongation or heading stage significantly reduced the grain yield, 1000-kernel weight and total biomass. Furthermore, kernels per spike and HI were significantly affected by water stress at elongation. There was no interaction between the two stages of treatment.

**Table 5:** The ANOVA table for yield, yield components, total biomass, and HI under drought/waterlogging stress at stages of elongation and heading.

Source of variation	Grain yield	Spikes per plant	Kernels per spike	1000-kernels weight	Total biomass	HI
Water stress at elongation (SSE)	35.9**	6.0	17.3*	20.5**	15.4*	54.1**
Water stress at heading (SHE)	12.9**	0.7	0.6	6.8*	7.9**	2.0
SSE×SHE	0.1	0.1	0.1	0.1	0.3	0.3

Values in the table are *F*-values.

* Significant difference at *P*≤0.05.

** Significant difference at *P*≤0.01.

#### Yield and yield components

Mild drought treatment during elongation did not significantly affect grain yield or yield components (Table 6). However, mild waterlogging stress significantly decreased grain yield due to the reduction of spike weight. Mild drought or waterlogging stress also significantly decreased grain yield, mainly due to 1000-kernel weight decrease.

**Table 6:** Yield, yield components, total biomass, and HI as affected by various degrees of drought and waterlogging stress.

Treatments	Grain yield (g plant^-1^)	Spikes per plant	Kernels per spike	1000-kernel weight (g)	Total biomass (g plant^-1^)	HI
Water stress at elongation						
Control	8.10a	3.80a	47.76a	44.61a	18.50a	0.39b
Mild drought	7.96a	3.70a	47.07a	45.67a	16.54b	0.42a
Mild waterlogging	6.91b	3.80a	42.97b	42.30b	17.74a	0.34c
Water stress at heading						
Control	8.20a	3.81a	46.59a	46.10a	18.39a	0.40a
Mild drought	7.19b	3.70a	45.92a	42.24b	16.80b	0.38a
Mild waterlogging	7.59b	3.79a	45.29a	44.23ab	17.59ab	0.38a

Different letters indicate statistical significance at the *P*≤0.05 level among different treatments at the same stages.

**Fig 4:**
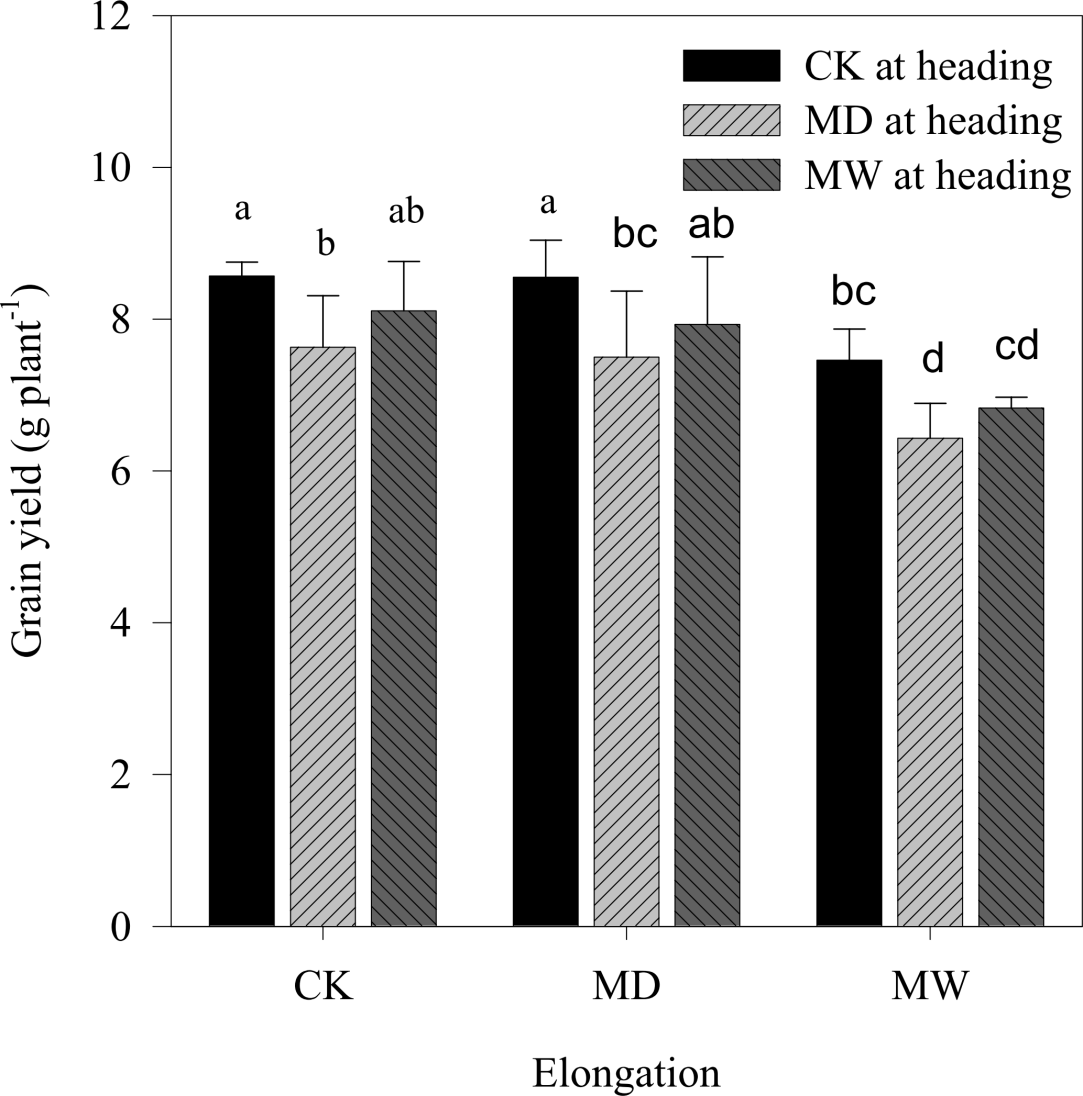
Effects of drought and waterlogging stress at the stages of elongation/heading on grain yield. CK, MD and MW denote control, mild drought and mild waterlogging, respectively. Different letters indicate statistical significance at the *P*≤0.05 level among different treatments.

Compared to the control values, no reduction was found in grain yield under the single mild drought treatment at elongation or the mild waterlogging stress at heading. However, it significantly decreased under single mild waterlogging stress during elongation and mild drought during heading, by 12.95 and 10.97%, respectively (Fig 4). The grain yield significantly decreased under cyclic water stress treatments, except for the combination of mild drought at elongation and mild waterlogging at heading. Further analysis indicated that grain yield under cyclic mild drought was significantly lower than that under the single mild drought at elongation; and under combinations of mild waterlogging at elongation and water stress at heading yield was much less than under the single treatments. Across all treatments, grain yield under a combination of mild waterlogging and mild drought was lowest, being 79.70% of the control treatments.

#### Total biomass and HI

The total biomass significantly decreased, whereas HI significantly increased or was not affected by mild drought either at elongation or at heading. Mild waterlogging at elongation significantly reduced HI (Table 6).

## Discussion

The effects of waterlogging and drought stress on wheat yield formation depend on the time stress occurs during the different growth stages. Most studies indicated that the reproductive phase, which is the phase from stem elongation to anthesis, was relatively less tolerant to water stress [6, 9]. Zhang et al. [4] found that wheat was more sensitive to drought stress from stem elongation to heading and heading to the milking stage. The results of the present study indicated that the yield reduction under either severe drought or severe waterlogging at elongation was more than at heading, whereas mild drought at elongation or mild waterlogging at heading did not significantly decrease the yield. Consequently, it has been demonstrated that the effects of water stress on wheat yield also depended on the stress severity. Several studies have revealed that mild drought stress even increased wheat yields [12, 13], whereas other studies have shown that mild drought dramatically decreased yield [6]. Moderate or severe drought stress, however, reduced grain yields, and this decrease was undoubtedly greater under severe stress [11, 13]. Consistently, in this study, yield losses increased as the degree of stress increased.

In general, water stress during the early growth stages mainly affected spikes, while kernels per spike and/or kernel weights were affected during the middle or late stages [26–28]. Araki et al. [7] found that waterlogging stress at elongation decreased the grain yield, mainly due to loss in the 1000-kernel weight. The present study showed that water stress at elongation decreased the spikes per plant and kernels per spike; however, drought stress increased the 1000-kernel weight, whereas waterlogging stress decreased the 1000-kernel weight. This different influence could be attributed to different injury degrees and destruction of sources and sinks by drought or waterlogging stress. Moreover, water stress at heading mainly reduced the kernels per spike and kernel weights. Previous studies have shown that the accumulation and remobilization of dry matter were influenced by water stress [29, 30]. This study indicated that both waterlogging and drought stress during the two stages reduced total biomass. An interesting result was that mild drought at elongation significantly increased HI and did not significantly decrease biomass. Similar results demonstrated that mild drought during all of the growth stages improved the distribution of carbon assimilate to the grain and increased the yield [13]. The other stress treatments decreased HI or did not affect HI.

Compared to the single water stress treatment, cyclic water stress is thought to be closer to natural conditions [23]. Single winter waterlogging or summer drought significantly decreased wheat yield, but the effects of the two stresses were not additive [26]. Previous study showed that single waterlogging caused a yield loss of 10–15% and 15% during elongation and anthesis stages, repectively; cyclic stress reduced the yield by 22~35% [7]. Izanloo et al. [23] suggested that drought-tolerant cultivars exhibited variation in yield loss when subjected to severe cyclic drought stress, but the differences were less pronounced under milder drought stress. The grain yield was not significantly affected by the combination of mild drought and mild waterlogging, mainly because wheat growth was only slightly influenced by the second treatment of a single water stress. Mild drought during elongation even improved the canopy structure before anthesis, maintained high canopy photosynthesis after anthesis, and increased the distribution of assimilate to grain, ultimately increasing wheat yield [13]. In general, a yield reduction from mild cyclic water stress does not mean there was more severe damage than from single treatments; in contrast, grain yield was damaged much more when water stress occurred again after severe drought and waterlogging. These experimental results were consistent with the previous conclusions under cyclic water stress, especially under cyclic severe drought or waterlogging treatments. According to previous theories, waterlogging occurring in early growth allows plants to recover from stress once the stress has been removed [8], but waterlogged plants had less time to recover when the stress occurred at a late growth stage. Gales et al. [31] considered that both the size of the root and the canopy were decreased by waterlogging, so that the decreased water uptake capacity was possibly balanced by the decreased transpiration area. The oxygen content in the soil and stomatal conductance of leaves could help plants to recover from the mild waterlogging stress, and vice versa when plants were subjected to extreme waterlogging stress [32]. Unlike waterlogging, drought generally causes a slow decrease in yield as the stress progresses; once water becomes available, significant recovery may occur [32]. However, the morphological and physiological recovery would be even slower or terminated when severe drought occurs. The effect of water stress will be overlaid when another water stress happens after the extreme waterlogging or drought stress.

The above results indicate that it is necessary to take stress severity into account when assessing the damage of water stress. The previous study confirmed that pre-anthesis short-period waterlogging priming could effectively alleviate yield loss under post-anthesis waterlogging in wheat, due to energy metabolism and stress defense induced by waterlogging priming to increase waterlogging tolerance [33]. This study did not observe a similar result, which could be attributed to the long stress duration resulting in reduction of leaf chlorophyll content, stomatal conductance or leaf water content, and photosynthetic decrease [7]. Although yield components, dry matter accumulation and remobilization account for the yield penalty, the physiological and molecular mechanisms of cyclic water stress that affect wheat remain unknown.

The changes in precipitation patterns have evidently affected agricultural production [34]. The phenomenon has become more apparent in recent years in the middle and lower reaches of the Yangtze River in China. According to meteorological and production statistics from Jiangsu province, in this region, drought and waterlogging stress frequently occur in spring, especially waterlogging, though the precipitation conditions in Jiangsu were generally at a perennial normal level. In northern China, however, water shortage is one of the main constraints influencing grain yield [13]. In addition, winter waterlogging and summer drought may become more prevalent as a result of climate change as suggested for the UK [17]. Compared to the persistent drought in northern China and seasonal waterlogging and drought in the UK, the reduction in yield potential due to cyclic waterlogging in the short term may be more serious, because of low-quality drainage systems. As a matter of fact, moisture excess led to more yield loss than moisture deficit in this region; in particular, extreme wetting in the late growth stages of wheat resulted in the most severe yield losses. Therefore, it is important to take corresponding measures, such as fertilization, for single water stress during early and middle growth stages. More importantly, prophylactic measures, such as improving drainage systems, should be provided for cyclic water stress because of limited recovery time.

## Conclusions

The study investigated the effects of single and cyclic water stress at the elongation and heading stages on yield, yield components, total biomass and harvest index of winter wheat. The single and cyclic water stress significantly decreased grain yield with the exception of mild drought at elongation and/or mild waterlogging at heading. The extent of yield loss increased with the increase in stress severity. Severe drought/waterlogging at elongation caused more yield damage than during the heading stage. In general, a yield reduction from mild cyclic water stress did not indicate more severe damage than from single treatments; in contrast, grain yield was damaged much more when water stress occurred again after severe drought and waterlogging. Across all treatments, cyclic severe drought and waterlogging reduced grain yield by 71.52 and 73.51%. The main reasons for the yield decrease could be summarized as: kernel number decrease due to drought at elongation, spike weight decreases due to drought at heading and waterlogging, as well as the decrease of total biomass and/or harvest index. The results provide important information for development of approaches to irrigation and drainage regulation to achieve high and stable wheat production.

## Supporting information

S1 Table Relative soil moisture content during experiments. (XLSX)
